# Health of Atlanta

**Published:** 1887-03

**Authors:** 


### Editorial.

### HEALTH OF ATLANTA.

The eighth annual report of the Board of Health of the city of Atlanta for the year 1886 has just been published and reflects great credit upon the gentlemen who constitute that body.

The maintenance of the public health is the most important uty of a city government, and it is gratifying to know that it has been entrusted to able hands. Every phase of sanitation has engaged the earnest attention of the board, and the report shows that all that careful, scientific investigation can suggest, or patient and wisely directed effort can accomplish, has been done to preserve the undoubted salubrity of the climate with which Providence has blessed us.

Statistical tables, which form part of the report (too long for insertion in THE JOURNAL), demonstrate that gratifying results have been accomplished. No epidemic of any disease has prevailed in the city; the number of deaths from diseases which may become epidemic by influences common in cities is annually diminishing. Notwithstanding the continued increase of our population, the astonishingly low rate of mortality encourages the expectation of even better things in the future.

We learn from the report that the total annual death-rate was 14.86 per thousand, estimating our population at 60,000—white 39,000, colored 21,000.

Of the total number of deaths there were 394 white and 498 colored. The annual rate of mortality per thousand among the whites was 10.10, among the colored population 23.71. The number of deaths of persons over five years of age reached 475—

white 234, colored 241. The deaths among children under five years of age were 417—white 160, colored 257. Of this number 231 were under one year old—white 79, colored 152. The number of deaths among children under five years of age for the four months of May, June, July and August was 219 against 198 for the other eight months of the year, and against 259 for the same months last year and 239 for the year before.

Among the principal causes of death, consumption stands charged with 119—white 49, colored 70; pneumonia 81—white 24, colored 57; other acute lung diseases 25—white 9, colored 16; diarrhœal diseases, including diarrhœa, enteritis, dysentery and cholera infantum, 192—white 81, colored 111. Of this number cholera infantum alone carried off 68—white 32, colored 36; and of this number, 42—white 21, colored 21—occurred in June, July and August against 26 in the remaining nine months of the year. Typhoid fever 29—white 10, colored 19. Malarial fever 6—white 1, colored 5. Scarlet fever 3—white 1, colored 2. Diphtheria 5—white 4, colored 1. Rheumatism 3—white 1, colored 2.

Twenty-nine deaths were due to accident and violence—21 white and 8 colored. There were 69 still-born children reported—34 white and 35 colored.

The greatest mortality among the whites occurred in the month of June when it reached 58. The lowest, in December, was 20. Among the blacks, the greatest mortality occurred in the month of June, 72; the lowest in November, 17.

It will become apparent by an analysis of these statistics that the total number of deaths, 46.74 per cent., occurred among children under five years of age. Consumption caused 13.34 per cent. of all deaths; acute lung diseases 11.87 per cent.; diarrhœal diseases 21.52 per cent., and typhoid fever 3.25 per cent.

Consumption, acute lung diseases and diarrhœal diseases caused 46.74 per cent. of all deaths. All other diseases 53.25 per cent.

